# Economic Evaluations of Mass Drug Administration: The Importance of Economies of Scale and Scope

**DOI:** 10.1093/cid/cix1001

**Published:** 2017-11-08

**Authors:** Hugo C Turner, Jaspreet Toor, T Déirdre Hollingsworth, Roy M Anderson

**Affiliations:** 1 Oxford University Clinical Research Unit, Wellcome Trust Major Overseas Programme, Ho Chi Minh City, Vietnam; 2 Centre for Tropical Medicine and Global Health, Nuffield Department of Medicine, University of Oxford United Kingdom; 3 London Centre for Neglected Tropical Disease Research, University of Oxford, United Kingdom; 4 Department of Infectious Disease Epidemiology, School of Public Health, Faculty of Medicine, St Mary’s Campus, Imperial College London, Norfolk Place, University of Oxford, United Kingdom; 5 Mathematics Institute, University of Oxford, United Kingdom; 6 School of Life Sciences, University of Warwick, Coventry, University of Oxford, United Kingdom; 7 Big Data Institute, University of Oxford, United Kingdom

**Keywords:** Neglected tropical diseases, costs, economic evaluations, mass drug administration

## Abstract

It is recognized that changing the current approaches for the control of the neglected tropical diseases will be needed to reach the World Health Organization’s (WHO) 2020 goals. Consequently, it is important that economic evaluations of the alternative approaches are conducted. A vital component of such evaluations is the issue of how the intervention’s costs should be incorporated. We discuss this issue—focusing on mass drug administration. We argue that the common approach of assuming an intervention’s cost per treatment is constant, regardless of the number of individuals treated, is a misleading way to consider the delivery costs of mass drug administration due to the occurrence of economies/diseconomies of scale and scope. Greater care and consideration are required when the costs are incorporated into such analyses. Without this, these economic evaluations could potentially lead to incorrect policy recommendations.

## Teaser

The common approach of assuming an intervention’s cost per treatment is constant is a misleading way to consider the delivery costs of mass drug administration. Greater consideration of how to incorporate costs into economic evaluations is needed.

The neglected tropical diseases (NTDs) are a group of chronic, disabling, and disfiguring conditions that are most prevalent in populations living in poverty [1]. These diseases cause a substantial health burden on poor populations in Africa, Asia, and Latin America, costing developing economies billions of dollars every year [1, 2].

Several of the most prevalent NTDs are controlled by preventive chemotherapy using mass drug administration, where treatment is given at a large scale to eligible populations within an endemic area, without testing the participants for the infection or performing individual diagnoses. Some treatment programs target specific age groups, such as school-aged children, whereas others target the whole community. There have been recent improvements in the progress made on the control of NTDs, with 1 billion people being treated for at least 1 NTD in 2015 alone [3]. It is important to note that these large-scale mass drug administration programs against NTDs are possible due to drug donations made by the pharmaceutical industry [4, 5].

Despite these achievements [3], it is recognized that changes in the current approaches will be required to reach the NTD control and elimination goals set by the World Health Organization (WHO) [4, 6]. When considering policies and their associated costs, it is important that economic evaluations of the potential alternative approaches are conducted in order to develop the most cost-effective policies. This is particularly important for NTDs, which are most prevalent in resource-poor settings. Furthermore, as the costs of NTD control gradually shift from international donors to the health budgets of endemic countries [4, 5], it will be important that ministries of health can evaluate the costs and cost-effectiveness of the different strategies in line with their health priorities.

It is common within economic evaluations to assume constant returns to scale, where the intervention’s unit cost (the cost per treatment) is constant, regardless of the number of people treated [7–12]. This implicitly assumes that the total cost of an intervention increases linearly with the number treated. However, this assumption can be inaccurate for costing certain interventions [7, 9–11, 13]. In this article, we discuss this issue of cost calculations within economic evaluations, focusing on mass drug administration and the importance of economies of scale and scope.

## ECONOMIES OF SCALE AND SCOPE

### Economies of Scale

**Figure 1.**
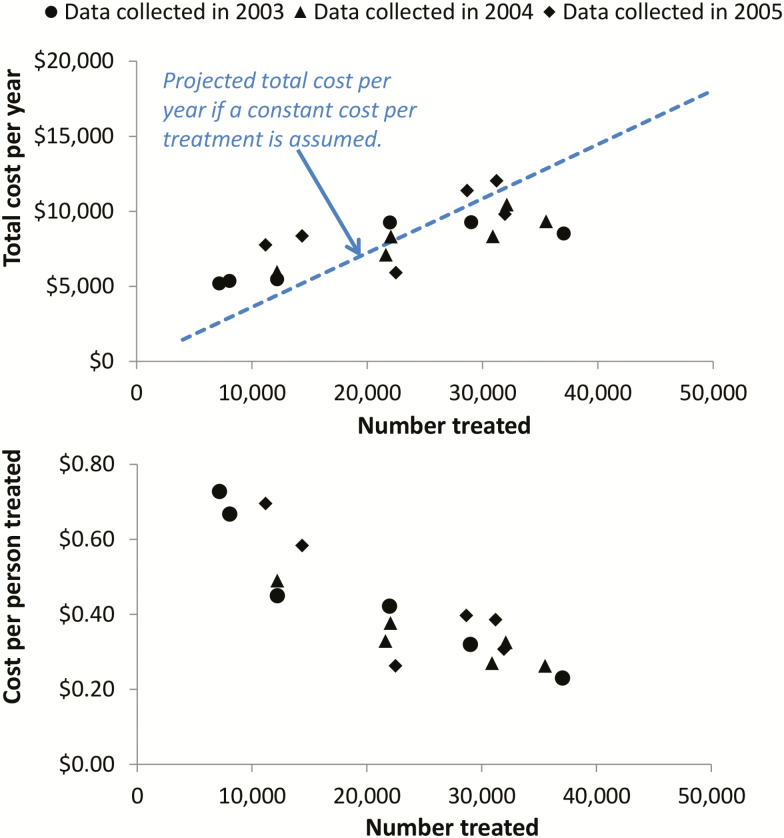
Economic cost of a school-based mass drug administration program using albendazole to target soil-transmitted helminths as a function of the number of children treated. The cost data were collected across six districts over three years, represented by the different markers [14]. The dotted line projects the total cost per year when assuming a constant cost per treatment (based on the average cost per treatment from this data set). The presented figures exclude the cost of praziquantel which is used to treat schistosomiasis. Costs are in 2005 prices.

Interventions such as mass drug administration can have strong economies of scale [11, 14–17] (box 1). This means that as the number of people treated increases, the cost per treatment decreases (Figure 1). This occurs because some of the costs associated with the delivery of mass treatment are fixed, that is, are incurred regardless of the number subsequently treated (Box 1), resulting in the fixed cost per treatment decreasing as the number treated increases. In reality, many of the costs will only be fixed for a particular level of activity and will increase incrementally once a threshold is crossed (stepped fixed costs), for example, the costs associated with needing to hire and train additional health workers when expanding into a new area or when their maximum capacity is reached (box 1). Due to the different types of fixed costs, there will be a difference in the economies of scale generated by increasing the number treated within a given operational area and increasing the number treated by expanding the operational area. Economies of scale can also occur over time as programs expand as they are likely to become more efficient through better organization and greater experience [7].

It is important to note that not all costs associated with mass drug administration will show economies of scale and some will scale directly with coverage (i.e., variable costs)—such as the costs associated with purchasing or storing treatment drugs (box 1).

Economies of scale are not unique to mass drug administration and can also occur for other interventions, such as vaccination [9], and within disease surveillance and monitoring and evaluation programs [18]. The reason they can be so significant for many of the NTDs is that the drugs are often donated and/or inexpensive resulting in the treatments delivery costs typically being the main driver in the programs overall cost. If the drugs were more expensive, it would increase the variable cost associated with each treatment; consequently, there would be less economies of scale. However, economies of scale can also occur when purchasing drugs with discounts offered for larger orders (see [19] for an example regarding the costs of malaria drugs).

### Diseconomies of Scale

It is possible for interventions to have diseconomies of scale [20]—where the average cost per treatment increases as the number treated is increased. This can happen when a program reaches full capacity leading to resources being overstretched and inefficiencies, or when expanding to populations which are more difficult to reach. For example, diseconomies of scale can occur when programs are expanded into rural areas as it has been found that the costs of health care interventions tend to be higher in rural settings than in urban settings [9, 21].

It is important to note that the presence of economies and diseconomies of scale are not mutually exclusive, and economic theory suggests that as the output of an intervention increases, its average cost per unit will first fall and then rise, resulting in a ‘u’-shaped average cost per unit curve [12, 20, 22–24]. For example, a regression analysis of 17 sex worker programs run by nongovernmental organizations in southern India found that the cost per commercial sex worker reached first decreased but then started to rise after the number reached exceeded around 1000–1700 [22].

However, in practice, this is not always the case and depending on the intervention and its location/cost structure, only economies or diseconomies of scale (or neither) may arise [12].

### Economies of Scope

NTD control programs have become increasingly integrated, targeting multiple diseases at once, as opposed to using separate disease-specific programs [25–27]. This integration is important, as it can result in economies of scope (box 1). In some cases, the diseases require the same drug (such as for river blindness and lymphatic filariasis, which both use ivermectin), whereas in others, different drugs are needed for each disease (such as for schistosomiasis and the soil-transmitted helminths). NTD control can also be integrated within interventions or programs targeting other diseases, generally at a low cost [11]. For example, treatment for the soil-transmitted helminths can be integrated into established immunisation or iron supplementation campaigns targeting adult women [28, 29], school-based vitamin A campaigns, and Child Health Days targeting preschool children [30].

As NTD programs progress, they may need to become increasingly integrated into other established programs, potentially moving from standalone campaign-style approaches to community or facility-based approaches.

### Why Considering Economies/Diseconomies of Scale and Scope is Important

**Figure 2.**
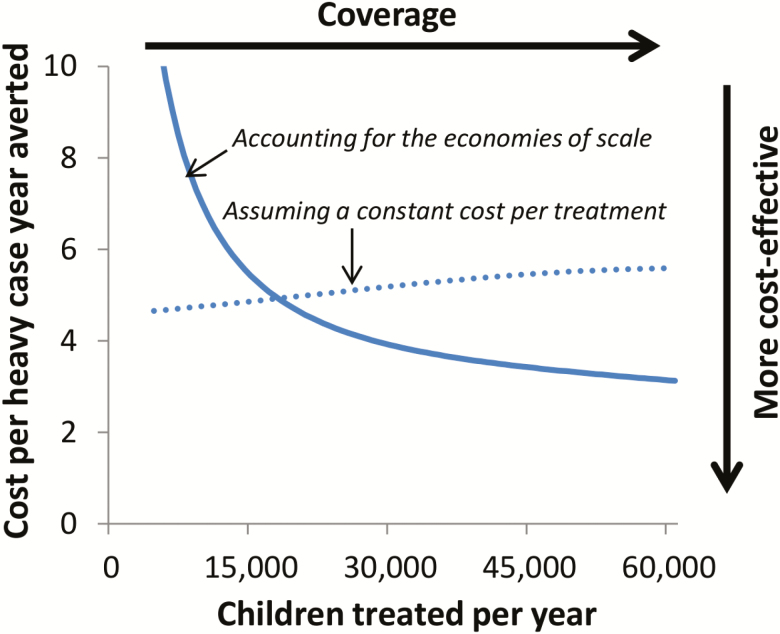
Projected cost-effectiveness of a school-based mass drug administration program targeting *Ascaris lumbricoides*. The cost-effectiveness decreases when assuming a constant cost per treatment because as the treatment coverage is increased, there is a degree of diminishing returns regarding the gains in effectiveness (in contrast, it is implicitly assumed that the total cost per year increases linearly with the number treated). The figure is adapted from Turner et al [13].

Due to economies of scale assuming a constant cost per treatment can be misleading for interventions such as mass drug administration. This is illustrated in Figure 1, which shows economic cost data collected from a school-based deworming program in Uganda [14] (the same trend can be seen in other data sets [15, 16], although this type of cost data is limited). These data clearly show that assuming a constant cost per person treated provides a poor representation of how the total cost per year of the program changes at different treatment coverage levels: underestimating the cost of treating a small number of people and overestimating the cost of treating a large number of people. A subsequent study incorporated this cost data into an economic evaluation using a soil-transmitted helminth transmission model [13]. It was found that when using a cost function that accounted for the economies of scale associated with the cost data, the model projected that the cost-effectiveness of mass drug administration would increase as the intervention was scaled up (Figure 2) [13]. However, if the economies of scale was ignored the opposite conclusion was observed with the cost-effectiveness being projected to decrease as the intervention was scaled up (Figure 2).

A summary of the key approaches that have been used been used to account for economies of scale in economic evaluations of mass drug administration are presented in box 2.

The presence of economies/diseconomies of scale and scope also has important implications regarding how generalizable an intervention’s cost per treatment is to other settings (both within and between countries) which subsequently affects the generalizability of economic evaluations guiding international health policy formulation. For example, due to economies of scale, it is possible that an intervention could only be cost-effective when performed at a large scale. It will also be important to consider the potential role economies of scope may have in influencing the costs of different NTD interventions [8, 27, 31] and the potential influence this could have on determining the most cost-effective intervention for a given setting [7].

Economies/diseconomies of scale and scope are also vital to consider when comparing the costs of different strategies/approaches. This is because economic evaluations of alternative interventions will often need to use cost data from different sources/studies. If the potential economies/diseconomies of scale and scope are ignored when doing this, analyses may make incorrect assumptions regarding the relative costs of different approaches, which could lead to the recommendation of suboptimal policies. This is particularly important given the recognised need for revised operational approaches in order to reach the WHO NTD goals [4].

The influence of economies/diseconomies of scale and scope will become increasingly significant as programs move toward the elimination goals. For example, due to economies of scale, as programs move toward elimination and stop treating in certain implementation units, their costs will not decrease linearly as there will be less economies of scale and scope, increasing the cost per treatment. This will be particularly important in light of the elimination goals for certain NTDs, as different diseases will progress to elimination at different speeds in co-endemic areas. For example, lymphatic filariasis control programs provide a platform that is leveraged to treat soil-transmitted helminths. Due to this, the average cost per person treated for soil-transmitted helminths is expected to dramatically increase as lymphatic filariasis programs are discontinued when geographic areas pass the transmission assessment survey [32, 33].

In the “last mile” or “end game” toward elimination, the treatment delivery costs may increase, as programs are expanded to cover harder-to-reach groups (diseconomies of scale)—such as in more remote rural areas [9, 21, 34]. The strength of these diseconomies of scale and the point at which they start to affect a control program will vary in different areas and to date little analysis of this issue has taken place. If these diseconomies of scale are ignored, the cost of the elimination strategies will be significantly underestimated. When performing economic evaluations of elimination strategies, the time horizon for the analysis will be particularly important—as this determines how long into the future potential cost savings are considered. A long-time horizon may be needed for the potential future cost savings associated with achieving elimination to counteract the increase in the interventions cost due to diseconomies of scale. It should be noted that several other variables are also significant when estimating the overall societal benefit of elimination, such as the projected productivity gains.

## CONCLUSIONS

Economic evaluations will play an increasingly significant role in informing NTD control strategies. More care and consideration are needed when the costs are incorporated into such analyses, particularly for interventions such as mass drug administration. The issues raised are also relevant for other large-scale intervention programs. Without considering the potential economies of scale and economies of scope associated with interventions, it is possible that cost data will be over generalised within economic evaluations, leading to poor policy formulation. Key requirements at present are a raised awareness of the need to obtain accurate cost data and the need to quantify the economies/diseconomies of scale and scope associated with health care interventions.

**Box 1: Glossary.**

**Diseconomies of scale:** The increase in the average cost per unit resulting from increased production/output, i.e. the opposite to economies of scale. This can result from programs expanding into harder-to-reach areas which can be more expensive.

**Economic costs**: The full value of all resources used for an intervention, including the value of donated resources for which no financial transaction has taken place. Economic costs are important when considering issues relating to the sustainability and replicability of interventions.

**Economies of scale**: The reduction in the average cost per unit resulting from increased production/output: in this case, the reduction in the cost per treatment that results from treating a larger number of people.

**Economies of scope**: The reduction in the average cost per unit resulting from producing two or more products at once: in this case, the reduction in the cost per treatment when delivering more than one intervention at once (i.e. integrated control programs). For example, administering treatment for both schistosomiasis and soil-transmitted helminths within the same program (as opposed to separate vertical programs for each disease).

**Fixed costs**: Costs that are not dependent on the quantity of output: in this case, costs that are incurred and do not change regardless of the total number of people treated. Examples of potential fixed costs for mass drug administration include many of the costs related to surveillance and the programmatic running costs incurred at a national level.

**Stepped-fixed costs**: Costs that are fixed for a particular level of activity/production, but increase incrementally once an activity threshold is crossed. For example, the costs associated with requiring another vehicle to deliver supplies and/or needing to hire and train additional health workers when expanding into a new area or when their maximum capacity has been reached.

**Variable costs**: Costs that vary in proportion to the quantity of output: in this case, costs that vary depending on the number of people treated. Examples of potential variable costs for mass drug administration include the costs associated with purchasing drugs, drug storage and the incidentals associated with providing treatment.

**Box 2: Solutions that have been used to account for economies of scale in economic evaluations of mass drug administration.**

In the following section, some of the past approaches that have been used to account for economies of scale in economic evaluations of mass drug administration are outlined. The optimal solution for a specific study will depend on the type of intervention that is being investigated and what cost data is available.

1. **Assuming the delivery costs are fixed for an area that is targeted for treatment:**
Several studies have assumed that the delivery costs of mass drug administration are fixed for an area that is targeted for treatment [35–38]. For example, the delivery costs for a control program targeting 80 communities are assumed to be essentially fixed for that area and not to change depending on the number of people who subsequently take the treatment within those targeted communities. In this case, even the economic value of the community health volunteers’ time can be effectively fixed for their community catchment area, as they will spend time visiting households regardless of whether the occupants take the treatment (e.g., some occupants may refuse treatment or be absent at the time of the visits).
A limitation of this approach is that it does not account for how the costs change when expanding treatment into a new area, that is, changing the geographical coverage. In addition, it does not account well for the fact that the achieved coverage and the costs of the program can be correlated (i.e. if an area invests more into community sensitization regarding the benefits arising from treatment, it can increase the coverage achieved). The latter limitation can be at least partly addressed by varying the costs assumed within any sensitivity analyses.
2. **Cost menus and micro-costing methods:**
Several studies have based their analysis on itemised cost menus (which list the quantity of each required resource, and their unit cost) [18, 39–44]. Depending on how these are developed, this approach can be effective at capturing a large proportion of the economies of scale associated with treatment distributed by mobile teams of health workers (the distribution method used at the time of many of those studies). This method has also been used for costing epidemiological surveys and screening strategies [18, 42]. However, for the current large-scale national programs, these simple cost menus are more difficult to define as teachers and/or community health volunteers/workers are used to distribute the treatments.
Kim et al [45], used a micro-costing method where they defined the key activities and resources required for onchocerciasis elimination and eradication (based on technical reports and budgets from the African Programme for Onchocerciasis Control). Though this approach was comprehensive, a limitation is that it is difficult to perform for programs/interventions that do not have such detailed technical reports and thorough program budgets. Furthermore, some economic costs may not be captured with this method.
3. **Cost functions:**
Turner et al [13] fitted a cost-function to the cost data from the school-based deworming program in Uganda shown in Figure 1. This captured how the costs of the program changed at different coverage levels. However, this approach requires detailed cost data to be collected at various stages of a program over multiple years—which is typically lacking for most countries with NTD control programs.
4. **Costing models:**
The WHO has recently developed a web-based regression statistical model which can estimate the delivery costs of mass drug administration [46, 47]. The model was based on a systematic review and a meta-regression of the obtained cost data. The model can estimate country-specific financial and economic delivery costs (with 95% confidence intervals) and can account for the economies of scale associated with mass drug administration. This model was subsequently used to perform an economic evaluation of the Global Programme to Eliminate Lymphatic Filariasis [48].
Kastner et al [49] also developed a micro-costing model, which was used to project the financial and economic cost of different lymphatic filariasis eradication scenarios.

## Notes

***Disclaimer.*** The views, opinions, assumptions, or any other information set out in this article are solely those of the authors. The funders had no role in study design, data collection and analysis, decision to publish, or preparation of the manuscript.

***Financial support.*** H. C. T. is supported by the Wellcome Trust [089276/B/09/7]. J. T., T. D. H., and R. M. A. gratefully acknowledge funding of the NTD Modelling Consortium by the Bill and Melinda Gates Foundation in partnership with the Task Force for Global Health and by the Children’s Investment Fund Foundation (UK).

***Potential conflicts of interests.*** R. M. A. is a nonexecutive director on the board of GlaxoSmithKline (GSK). GSK played no role in funding this research nor in the production of this paper. All authors have submitted the ICMJE Form for Disclosure of Potential Conflicts of Interest. Conflicts that the editors consider relevant to the content of the manuscript have been disclosed.
